# Origin of chromic effects and crystal-to-crystal phase transition in the polymorphs of tyraminium violurate

**DOI:** 10.1107/S2052252518017037

**Published:** 2019-01-24

**Authors:** Marlena Gryl, Agnieszka Rydz, Joanna Wojnarska, Anna Krawczuk, Marcin Kozieł, Tomasz Seidler, Katarzyna Ostrowska, Monika Marzec, Katarzyna Marta Stadnicka

**Affiliations:** a Faculty of Chemistry, Jagiellonian University, Gronostajowa 2, 30-387 Kraków, Poland; b Institute of Physics, Jagiellonian University, Łojasiewicza 11, 30-348 Kraków, Poland

**Keywords:** chromic effects, crystal engineering, crystal-to-crystal phase transitions, polymorphism, birefringence, tyraminum violurate

## Abstract

Chromic materials are nowadays widely used in various technological applications, however understanding the effect and the possibility of tuning the obtained colour of a material are still challenging. Here a combined experimental and theoretical study is presented on the solvatochromic and crystallochromic effects in the (pseudo)polymorphs of tyraminium violurate. This organic material exhibits a large solvatochromic shift (*ca* 192 nm) associated with broad colour change (from yellow to dark violet). Tyraminum violurate crystallizes as red crystals of form (I) from water as a solvate, and as an unsolvated form [violet crystals of (II)] from methanol solution. Form (I), when heated, undergoes two crystal-to-crystal phase transformations associated with colour change of the crystals. Crystals of (II) show extreme birefringence (*ca* 0.46) and high refractive index (*n*_γ_ above 1.90), which can be correlated with preferential orientation of the resultant dipole moments of the ions. Examination of optical effects (UV–Vis spectra) along with theoretical calculations (QTAIM, atomic and bond polarizabilities) enabled the description of the origin of colour in the studied materials.

## Abstract 2

A new approach to understanding the chromic properties of crystals was tested on (pseudo)polymorphs of tyraminium violurate with large solvatochromic and crystallochromic effects, extreme birefringence, and crystal-to-crystal phase transition.

## 1. Introduction   

Over the years materials capable of stimulated colour change (chromic effect) found vast applications in science and technology (Hutchins *et al.*, 2014 ▸; Bašnec *et al.*, 2018 ▸; Bamfield & Hutchings, 2018 ▸). Selective colour change is nowadays used in multiple devices *e.g.* photochromic lenses, smart self-dimming windows, thermal papers, paints and indicators, visual displays or biochemical probes. Chromic phenomena can be classified based on the source of an external stimulus, which can be irradiation (photochromism), mechanical force or pressure (mechanochromism), electric field (electrochromism), heat (thermochromism), solvent (solvatochromism) or aggregation effects (crystallochromism). The origin of the colour change can be associated with one of the five factors (Nassau, 1987 ▸): (1) vibrations and simple excitations, (2) ligand-field effects, (3) electron transfer between the orbitals, (4) transition between energy gaps, and (5) geometrical and optical effects *e.g.* interference, iridescence, diffraction. This is a simplified classification as the relationship between the observed colour and the geometric arrangement of the molecules in the solid state or in solution is still not fully understood. It is known that the existence of weak interactions between molecules has a significant impact on effects such as solvatochromism or a solid-state crystallochromic effect (Marini *et al.*, 2010 ▸; Li *et al.*, 2015 ▸; El-Sayed *et al.*, 2003 ▸). Solvatochromism is based on the differences in solvation energies of the ground and excited states which is reflected in shifts of the absorption or emission maxima of the solvated compound. The influence of the solvent on the observed spectra is described using the ‘solvent polarity’ concept (Reichardt, 1994 ▸). This ‘polarity’ differs from the classical definition and can be associated with solvent dipolarity, polarizability or hydrogen-bonding properties. In general, when the excited state of a molecule is more polar than the ground state, the more polar solvents favour the stabilization of the excited state and a bathochromic shift can be observed (positive solvatochromism) (Bamfield & Hutchings, 2018 ▸). On the other hand, a more polar ground state is the cause of an opposite effect and a hypsochromic shift can be observed in the spectrum (negative solvatochromism). The crystallochromic effect can be even more complex in nature as it is directly associated with the changes in molecular structure that are also dependent on aggregation in the solid state. It is especially evident when different polymorphic forms of a material are found and the colour change can be directly correlated with a particular crystal packing (Yu *et al.*, 2000 ▸; Chen *et al.*, 2005 ▸). This definition excludes the specific case of colour change of a material caused by conformational change of a molecule (Fujimoto & Kitamura, 2013 ▸). In practice, the origin of colour change is believed to be associated with the shift of electron density from one part of the molecule to another, often as a result of intermolecular interactions (Kitamura *et al.*, 2010 ▸). Typically the colour of pigments is explained on the basis of existing solid-state π-stacking interactions (Würthner *et al.*, 1999 ▸; Kazmaier & Hoffmann, 1994 ▸). In multicomponent materials, the solid-state aggregation process can be more complex and chromophore dependent, thus it is often difficult to predict the interactions and to what extent they will impact the absorption. Despite the vast literature on chromic effects (Błasiak *et al.*, 2017 ▸; Dzesse *et al.*, 2018 ▸; Kolev *et al.*, 2009 ▸; Koleva *et al.*, 2010 ▸), there is no general recipe which would allow explanation of the origin of colour, as well as control the effect.

In this article, we present an innovative approach towards understanding of colour in the solid-state polymorphs. The material designed in our group and chosen for this study – tyraminium violurate – is an example of how chromic effects can be tuned through co-crystallization. Both components, tyramine (TYR) and violuric acid (VA), are colourless solids and only when combined form colourful multicomponent systems. Multicomponent materials containing neutral violuric acid molecules (ketone–oxime form) either lack colour or form *e.g.* light yellow or orange crystals. The more acidic nitroso–enol form of violuric acid is believed to be formed in solution prior to a reaction between violuric acid and a base (Dass & Dutt, 1939 ▸). The intense colours of the formed salts (Banik *et al.*, 2016 ▸; Illán-Cabeza *et al.*, 2011 ▸, Kolev *et al.*, 2009 ▸) have been experimentally ascribed to the *n*→π* transition in the violurate ion (Awadallah *et al.*, 1994 ▸). The energy of the *n*-orbital of the nitro­gen atom and the π*-orbital delocalized over the anion can be affected by intermolecular interactions and the energy of the *n*→π* transition changes. In this way the exact position of the absorption maxima and thus different colours of the obtained materials are directly dependent on the intermolecular interactions involving violurate ions. Considering possible mixing of the *n*-orbital of the oxime nitro­gen atom with the adjacent oxygen atom lone pair, we should examine all the interactions involving the oxime group in violurate ions to understand the change in the absorption spectra. [Chem scheme1] 
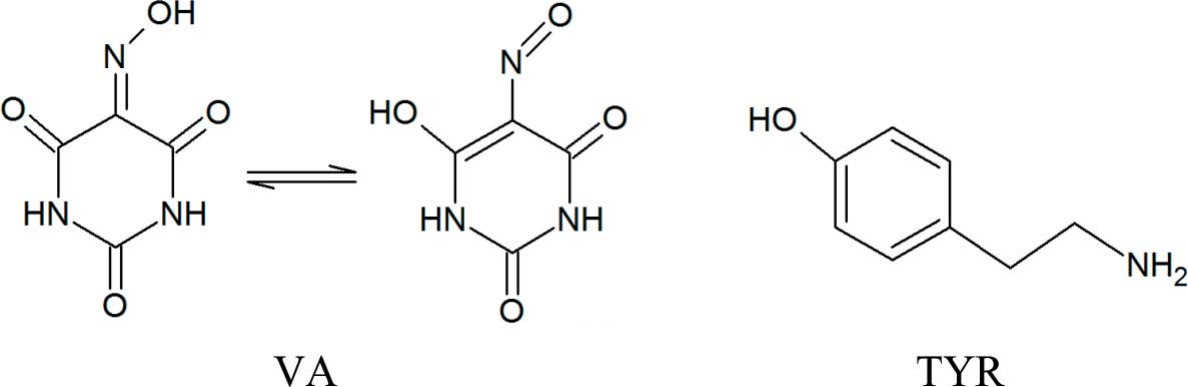


Polymorphs and a pseudopolymorph of tyraminium violurate salt, presented in this article, are suitable for verifying the theory that the colour is a result of a salt formation (substrates are colourless) and is not directly dependent on crystallization conditions (concomitant polymorphism). Moreover, the observed crystallochromic effects are far from typical – in the examined tyraminium violurates there are no π⋯π or π-stacking interactions. To correlate the colour with specific interactions, all crystal phases were extensively analysed using experimental techniques such as X-ray diffraction, UV–Vis spectroscopy for solid-state and liquid samples, differential scanning calorimetry (DSC), and measurements of refractive indices. The topology of electron density (QTAIM) (Bader, 2003 ▸), atomic and bond polarizabilities as well as refractive indices were studied in terms of quantum-mechanical calculations to clarify the mechanism of crystallochromy in the examined materials. To the best of our knowledge this is the first attempt to understand the origin of colour in a solid-state system by means of a combined topological analysis of electron density and atomic/bond polarizabilities approach (Macchi & Krawczuk, 2015 ▸).

## 2. Experimental   

### 2.1. Crystal engineering   

The mutual arrangement of molecules in the crystal structure is known to influence the physical and chemical properties of a material (Aakeröy *et al.*, 2015 ▸; Desiraju, 2014 ▸; Gryl *et al.*, 2015 ▸). It is a fact that the probability of obtaining a multi-component material increases for building blocks with synthon formation flexibility (Du *et al.*, 2006 ▸; Gryl *et al.*, 2008 ▸). The different utilization of hydrogen-bond donor and acceptor sites is also a reason for obtaining polymorphic systems of multicomponent materials. Both building blocks, violuric acid and tyramine, were chosen based on their structural complementarity. Tyramine [4-(2-amino­ethyl)­phenol] is a known neurotransmitter used for example in the treatment of Parkinson’s disease (Cruickshank *et al.*, 2013 ▸). Violuric acid, 2,4,5,6(1*H*,3*H*)-pyrimidine­tetrone-5-oxime, is an iso­nitroso derivative of barbituric acid with two reported pseudopolymorphic monohydrate forms (Guille *et al.*, 2007 ▸). Barbiturates are known to form multicomponent materials, showing interesting optical effects, when combined with suitable co-formers (Gryl *et al.*, 2013 ▸, 2015 ▸, 2018 ▸; Gryl, 2015 ▸).

**Figure 1:**
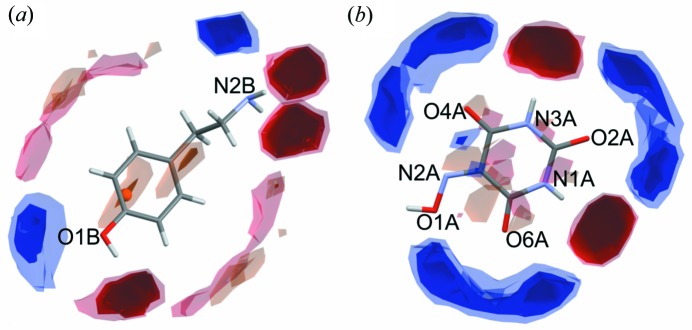
Full interaction maps of (*a*) TYR and (*b*) VA. Blue denotes possible donor sites, whereas red denotes possible acceptor sites.

The compatibility of the donor and acceptor groups of both molecules was predicted using combined Full Interaction Maps (FIM) (Wood *et al.*, 2013 ▸) and transferable synthon analysis (CSD search). FIM is a statistical tool used to visualize probabilities of finding donor (blue colour) and acceptor sites (red colour) or predict other weak interactions (light orange) in the proximity of a selected molecule. The FIM map for TYR [Fig. 1 ▸(*a*)] shows that amine and hydroxyl groups will most probably participate both as donors and acceptors of hydrogen bonds. In the case of VA [Fig. 1 ▸(*b*)], all oxygen atoms are potential acceptors of hydrogen bonds. N1A and N3A nitro­gen atoms can be considered as good donors, whereas the N2A nitro­gen atom is likely to be an acceptor of a hydrogen bond. Hydrogen-bond synthons found in the crystal structures of TYR (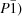, and in two polymorphs of violuric acid monohydrate [(I), *P*2_1_/*n*, (II), *Cmc*2_1_] confirm the FIM findings (for more details, see Fig. S1 in the Supporting information).

**Figure 2:**
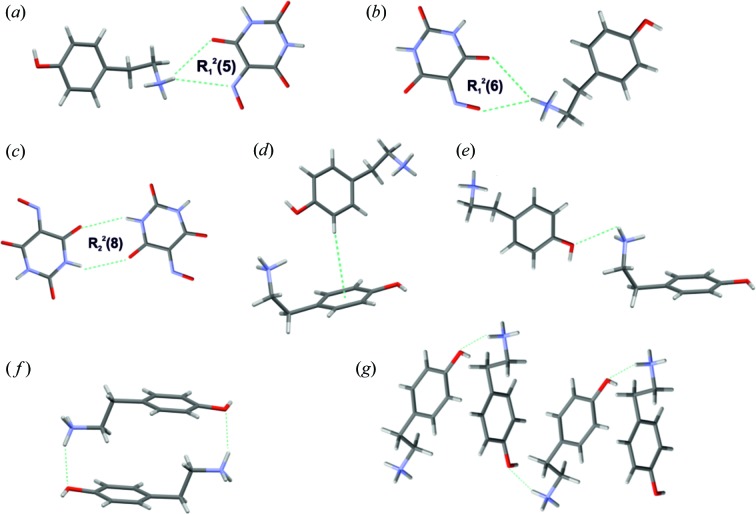
(*a*)–(*g*) Possible hydrogen-bond motifs to be formed between tyraminium cations and violurate anions. Figure prepared in Mercury 3.10.2 (Macrae *et al.*, 2008 ▸).

**Figure 3:**
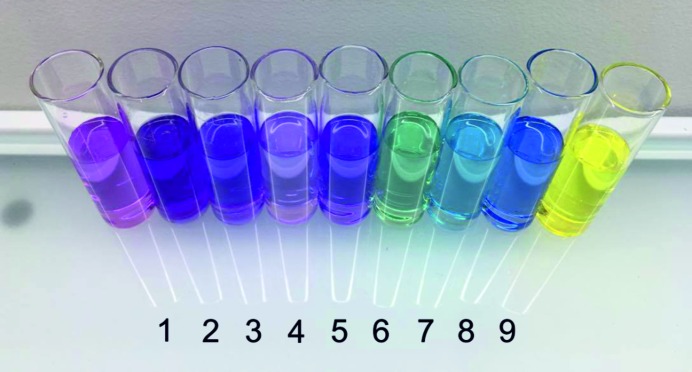
Solutions of tyraminium violurates in (1) water, (2) methanol, (3) ethanol, (4) 1-butanol, (5) 2-propanol, (6) DMSO, (7) DMF, (8) pyridine and (9) 1,4-dioxane.

Taking into account both synthon transferability and the acidic/basic character of the building blocks, we have predicted the probable motifs in the obtained crystal structures (Fig. 2 ▸). The affinity of violuric acid to water was taken into account by introducing this solvent as a connector in some of the designed motifs. The crystallization experiments were designed accordingly. Tyraminium violurate crystals were obtained by mixing equimolar saturated solutions containing tyramine (Sigma-Aldrich) and violuric acid monohydrate (Sigma-Aldrich). Red crystals of (I) were obtained when water was used as a solvent whereas violet crystals of (II) grew from methanol solution. In both cases mixing substrates resulted in a dark purple solution, which was left for a few days at room temperature for slow evaporation. After a couple of days violet crystals also appeared in the water solution (concomitant polymorphs). Phase (III) was the result of a solid–solid phase transition: crystals of monohydrate form (I) were heated up to 160°C for 3 h (both single crystals and powdered samples) and left for slow cooling for 12 h. Tyramine and violuric acid in 1:1 ratio were also combined in a series of 15 solvents to examine the possible solvatochromic effects. Despite the fact that all solutions were tightly closed in vials, in some of them instant crystallization was observed, and because of that only nine clear solutions were used for further analysis (Fig. 3 ▸).

### 2.2. Data collection and refinement   

Single crystals of (I), (II) and (III) suitable for X-ray diffraction analysis were selected under a polarizing microscope. Single-crystal X-ray diffraction experiments were performed on a Rigaku SuperNova diffractometer at 130 K. All data were integrated with *CrysAlisPro* (Rigaku Oxford Diffraction, 2015 ▸). Crystal structures were solved using direct methods in *SIR97* (Altomare *et al.*, 1999 ▸) and refined using least-squares procedures in *SHELXL-2017/1* (Sheldrick, 2015 ▸). Details of data collection and refinement are presented in Table S1.

### 2.3. DSC and powder X-ray diffraction measurements   

Calorimetric measurements were performed using a Perkin Elmer Diamond 8000 differential scanning calorimeter. The sample of (I) (5.21 mg) was placed in an aluminium crucible and firmly closed with a press. The measurements were carried out in the temperature range 25–200°C; at first the sample was heated and then subsequently cooled down at rate equal to 5°C min^−1^. Powder diffraction patterns of all studied samples were collected using a PANalytical X’pert PRO MPD diffractometer in Bragg–Brentano geometry. The investigations were carried out at ambient pressure. A copper X-ray source (λ = 1.5418 Å) was used for all experiments. The measurement temperature was controlled with an Anton Paar TTK-450 low-temperature chamber.

### 2.4. Refractive indices measurements   

The refractive indices of crystals (I) and (II) were determined by the immersion method using a Zeiss Axio ScopeA1 polarizing microscope. Crystals were placed on a glass plate under a cover slip and immersed in a mixture of liquids with known refractive indices. Samples prepared in this way were observed under the polarizing microscope at large magnifications (200×). Liquids used in the experiment were a mixture of xylene isomers with the refractive index 1.449, bromo­form (1.589), methyl iodide (1.740) and a supersaturated solution of sulfur in methyl iodide (1.82) (see Table S2). The match between the refractive index of a liquid and that of a crystal was judged by the observation of the Becke lines. These bright halo contours created at the crystal liquid borders move towards the medium of higher refractive index when the stage of the microscope is lowered. A match between the refractive index of a crystal and of the liquid causes the Becke lines to disappear.

### 2.5. UV–Vis measurements   

UV–Vis spectra for tyraminium violurate solutions were recorded using a Hitachi U-3900H spectrophotometer in 1 cm cells at 25°C after equilibrating for 20 min. UV–Vis measurements for ground tyraminium violurates crystals mixed with barium sulfate were performed using the 50 mm transmission/reflectance sphere on PerkinElmer LAMBDA 365 Spectrophotometer at room temperature.

### 2.6. Theoretical calculations   

The wavefunctions for calculations of theoretical electron density were obtained using the periodic B3LYP/POB-TZVP method with *Crystal17* software (Dovesi *et al.*, 2018 ▸, 2017 ▸), for experimentally determined geometries of (I), (II) and (III). Additionally, periodic calculations were performed for a chosen polymorphic form of VA (*Cmc*2_1_). Topological properties according to QTAIM were studied using the program *TOPOND* (Gatti *et al.*, 1994 ▸; Frisch *et al.*, 2016 ▸). *Ab initio* calculations [*GAUSSIAN16* (Gatti & Casassa, 2013 ▸) package at B3LYP/TZVP level] were performed for the isolated molecules and for clusters in which all possible intermolecular interactions of the oxime group were included. Geometries were taken from experimental X-ray diffraction data and kept frozen. Cluster wavefunctions were further used to calculate the source function (Gatti, 2012 ▸) and interaction energies, with the use of *AIMAll* package (Keith, 2017 ▸). Additionally, in order to obtain information on atomic and bond polarizabilities, wavefunctions were also generated at zero field as well as under a small (0.005 a.u.) electric field directed along ±*x*, ±*y* and ±*z*, and further used for QTAIM partitioning with the *AIMAll* package. Atomic polarizability tensors were then estimated, using *PolaBer* software (Krawczuk *et al.*, 2014 ▸), as numerical derivatives of the electric dipole moment with respect to the external electric field. The local field theory (LFT) approach was used to calculate the refractive indices of (I) and (II) (Seidler *et al.*, 2014*a* ▸,*b* ▸, 2015 ▸, 2016 ▸). The positions of atoms were optimized at fixed cell parameters using *Crystal14* (Dovesi, Orlando *et al.*, 2014 ▸; Dovesi, Saunders *et al.*, 2014 ▸) at B3LYP/6-31G** level of theory. For (I), only one oxime group position was selected. The molecular polarizability calculations were performed using MP2/6–311++G(d,p) (static values; λ = ∞) applying a charge of −1 for the violurate anion and +1 for the tyraminium cation. The frequency dispersion was introduced with MP2 [B3LYP/6–311++G(d,p)] static and dynamic polarizability tensors with the use of the approach described in the literature (Seidler & Champagne, 2016 ▸).

## 3. Results and discussion   

### 3.1. Crystal structure description   

**Figure 4:**
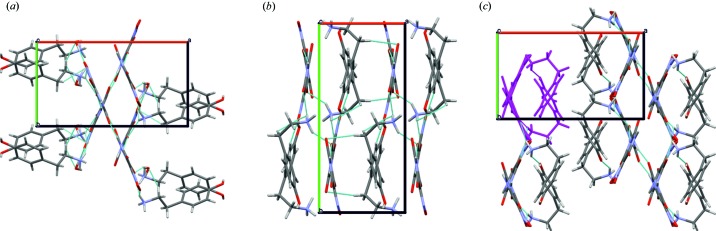
Packing of the molecules in the crystals of (*a*) (I), fragment of the unit cell (0,1/2*c*), (*b*) (II), and (*c*) (III), all along the [001] direction. Heart-shaped motifs are marked in violet.

Crystals of (I) belong to the monoclinic crystal system (*P*2_1_/*c*), with one anion of violuric acid, one cation of tyramine and one water molecule in the asymmetric unit [see Fig. S2(*a*)]. The oxime group is disordered over two positions in 0.76:0.24 ratio. Violurate anions are arranged in centrosymmetric dimers via N-H⋯O hydrogen bonds. The crystal structure of (I) is built of alternate layers constructed from either violurate anions or tyraminium cations. In the anionic layer violurate ions are arranged in a herringbone motif. Water molecules act as linkers forming hydrogen bonds interconnecting both types of ions. The analysis of weak interactions in the crystal structure of (I) confirmed the existence of 11 crystallographically independent hydrogen bonds (see Table S3) responsible for the observed dense hydrogen-bonds network [Fig. 4 ▸(*a*)].

The structure of (II) follows the symmetry of space group *P*2_1_/*c* and contains one tyraminium cation and one violurate anion per asymmetric unit [see Fig. S2(*b*)]. Violurate anions form crinkled tapes along the **c** direction. The tapes are connected via tyraminium cations by N—H⋯O and O—H⋯N hydrogen bonds [Fig. 4 ▸(*b*), Table S4]. Characteristic 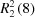 dimers (see graph-set theory in Etter *et al.*, 1990 ▸) between violurate anions are present in the crystal structures of (I) and (II). Larger rings of 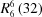 type, built of violurate ions, and 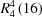, formed using both types of ions, are present in (I) and (II), respectively (see Figs. S3 and S4). Crystals of (III) were obtained from (I) as a result of crystal-to-crystal phase transition. These crystals are dark red with metallic lustre and have clearly visible cracks which are the result of heating. The structure follows the symmetry of the *P*2_1_/*c* space group. The asymmetric unit [see Fig. S2(*c*)] contains two ions in a 1:1 ratio as found in (II). Nitro­gen and oxygen atoms of the oxime group are disordered over two positions in a 0.54:0.46 ratio. The number of crystallographically different hydrogen bonds is increased from eight to ten in contrast to polymorph (II) – see Table S5. Characteristic 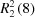 motifs and larger 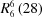 and 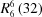 rings are also present in this structure. The crystal structure of (III) is composed of alternate layers of tyraminium cations and violurate anions connected through weak C—H⋯π interactions between tyraminium cations (see Fig. S5), in contrast to (I) where layers are connected via hydrogen-bond interactions with water molecules. Tyraminium cations in all three structures differ in conformation (see Fig. S6). The torsion angle of the side chain C4B—C7B—C8B—N2B in (I) equals almost 180°, which can be associated with an antiperiplanar conformation. In (II) the same angle is *ca* 68°, which is closer to a gauche conformation. The phase transition from (I) to (III) causes the rotation of the NH_3_^+^ group around the C7B—C8B bond and the resultant torsion angle is 55°. The tyraminium ions in both polymorphs (II) and (III) also differ in the orientation of the O—H group (rotation of about 157° around C1B—O1B bond). Fig. 4 ▸(*c*) shows heart-shaped motifs formed between violurate anions and tyraminium cations in (III) caused by the specific conformation of tyraminium cations. It is worth noting that in all of the examined polymorphs there are no π⋯π interactions (for more details please see Figs. S7, S8 and S9).

In order to reveal the factors that contribute to spatial arrangement of intermolecular interactions, Hirshfeld surface (HS) and fingerprint plot (Spackman & Jayatilaka, 2009 ▸; Spackman & McKinnon, 2002 ▸) analyses were performed. The percentage of interactions was determined through the fingerprint plots. The results for tyraminium cations and violurate anions are presented in Figs. S10 and S11, respectively. Fingerprint plots for anions of violuric acid and cations of tyramine can be interpreted in a similar way. The main difference between (I), (II) and (III) is the percentage of O_in_⋯H_out_ and H_in_⋯O_out_ interactions (‘in’ denotes the atom inside the HS and ‘out’ denotes the atom outside the HS). In (I), the presence of water molecules leads to stronger hydrogen bonds formed between violurate anions and water molecules which increases the percentage of O–H interactions to 59.7%. In the two remaining structures this percentage is slightly smaller: 56.3% in (III) and 53.6% in (II). A similar situation can be observed for tyraminium cations, where H_in_⋯O_out_ and O_in_⋯H_out_ interactions in (I) are stronger than in (II). The O_in_⋯H_out_ and H_in_⋯O_out_ interactions are marked by two sharp spikes located at the bottom of the drawing. In (II), numerous, dispersed points between the two spikes refer to H⋯H contacts, whereas in (I) the same interactions are located in the middle of the plot. In (II) the percentage of C_in_⋯H_out_ and H_in_⋯C_out_ interactions involving violurate is three times higher than in (I) and (III). All of those distinct weak interactions are responsible for different packing motifs in the examined structures observed in spite of the same global symmetry elements of the *P*2_1_/*c* space group.

### 3.2. Crystal-to-crystal phase transition and optical properties   

**Figure 5:**
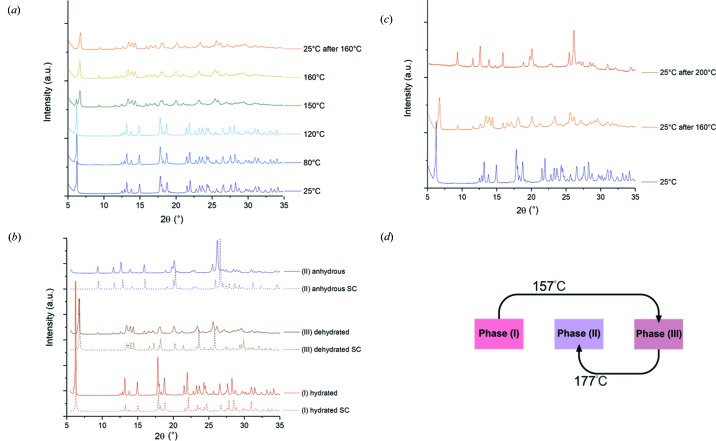
(*a*) Temperature evolution of (I) – phase transformation of (I) to (III) at *ca* 150°C. (*b*) X-ray powder diffraction patterns of (I), (II) and (III) (at room temperature), SC – simulated pattern from single-crystal X-ray diffraction. (*c*) Comparison between form (I) at 25°C, which was then transformed to (III) at 150°C and finally to (II*a*) at *ca* 200°C. (*d*) Scheme of the phase transition between the polymorphs.

Fig. S12 presents DSC curves registered for heating and cooling of (I). Heating the sample results in two anomalies. The strong exothermic peak appearing with a minimum at 157°C (Δ*H* = −992 J g^−1^) is related to dehydration of (I) and to the first phase transformation to (III). In turn, the weak endothermic anomaly registered with a maximum at *ca* 177°C (Δ*H* = 80 J g^−1^) is associated with a second phase transformation in the structure of the studied crystals. During cooling no anomaly was recognized, which means that processes registered during heating are irreversible. Fig. 5 ▸(*a*) shows the evolution of the powder diffraction pattern of (I) at different temperatures. At *T* = 25, 80 and 120°C the same phase of (I) is observed. At 150°C the intermediate state was captured, while at 160°C a new pattern matching the pattern of (III) is observed. The new form (III) does not return to its original state upon cooling back to room temperature, which confirmed the irreversible character of the transition from (I) to (III) at 157°C. Subsequent heating of the sample to 200°C triggers the second, endothermic transition yielding another powder pattern, matching the pattern of (II) – we will refer to this sample as (II*a*). This pattern also does not change on cooling to room temperature. Powder patterns of (I), (II) and (III) match those generated from single-crystal data [Fig. 5 ▸(*b*)]. Minor differences in peak positions between collected and respective simulated patterns are attributed to different measurement temperatures for powder and single-crystal experiments. The powder pattern of (III) exhibits a strong resemblance to the simulated one; however, it also includes weak signals matching the form (II). Partial transformation to (II) occurs because of minor irregularities in the heat distribution during powder diffraction measurements. The transition temperatures derived from powder diffraction data are consistent with the DSC experiment, taking into account the scanning speed of the DSC measurement in relation to pattern collection time. Fig. 5 ▸(*c*) presents a comparison between form (I) at 25°C, which was then transformed to (III) at 150°C and finally to (II*a*) at *ca* 200°C. The scheme of all phase transformations is presented in Fig. 5 ▸(*d*).

**Figure 6:**
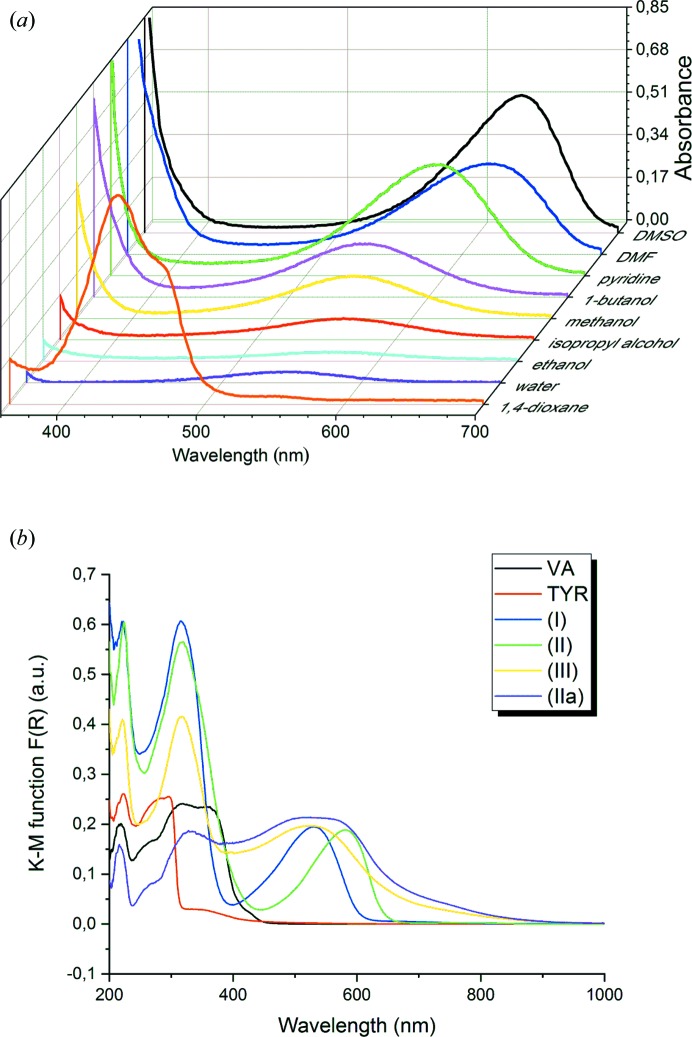
UV–Vis spectra of tyraminium violurate (*a*) in selected solvents, and (*b*) in solid-state samples of (I), (II), (III) and (II*a*) in comparison with TYR and VA monohydrate.

UV–Vis spectra for tyraminium violurate solutions in various type of solvents are illustrated in Fig. 6 ▸(*a*). All solutions prepared with the use of aprotic solvents (1,4-dioxane, pyridine, acetone, DMF and DMSO) were chosen to determine the character of the solvatochromic effect. Tyraminium violurate solutions demonstrate a positive solvatochromic effect equivalent with the bathochromic shift from λ_max_ = 438.15 nm in 1,4-dioxane to λ_max_ = 630.81 nm in DMSO (Δλ_max_ = 192.66 nm).

UV–Vis spectra of crystalline phases (I), (II), (III), (II*a*), VA and TYR were also examined [Fig. 6 ▸(*b*)]. The Kubelka–Munk function was used to obtain diffuse reflectance spectra. The real colour of the crystals, (I) red and (II) violet, corresponds to the positions of maxima: λ_max_(I) = 530 nm (green region), λ_max_(II) = 581 nm (yellow region), respectively. In the case of (III) there is a broad maximum in the visible range with λ_max_ = *ca* 524 nm which corresponds well with the red–brown colour of the sample. The (II*a*) sample was obtained from (I) after heating to 200°C and cooling to room temperature. The obtained UV–Vis spectrum for (II*a*) is different from that of form (II), which is not entirely surprising. The heating process and phase transition affected the quality of the material obtained; it introduced defects that caused a change in colour with respect to phase (II) obtained at room temperature. Based on the position of maxima, transition energies are equal to 2.33, 2.13 and 2.35 eV for (I), (II) and (III), respectively.

**Figure 7:**
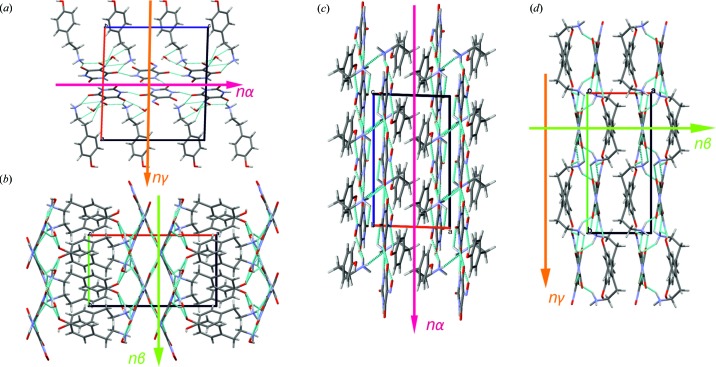
Correlation between directions of optical indicatrix axes (refractive indices) and crystallographic axes in the structure of (I) (*a*, *b*) and (II) crystals (*c*, *d*).

Crystals of (I) and (II) of optical quality were selected for refractive indices measurement using the immersion oil method. Table 1 ▸ contains a comparison of experimental and selected theoretical values of the refractive indices of crystals (I) and (II) (the full data are available in Table S6). The directions of the optical indicatrix with respect to the crystallographic axes were determined using *CrysAlisPro* (Rigaku Oxford Diffraction, 2015 ▸) (see Fig. S13). Crystals of (III) were not transparent and this fact excluded them from refractive indices measurements. Crystals of (I) are optically biaxial with 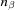 along the unique *b* axis. The two remaining refractive indices are located in the *ac* plane, with 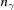 almost parallel to the [100] direction and 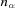 close to the [001] direction. Crystals of (II) are also biaxial with 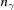 in the [010] direction. The 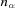 and 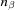 are located in the *ac* plane and deviate a few degrees from the [001] and [100] directions, respectively (Fig. 7 ▸). The 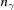 for (II) was not determined directly as the value exceeded 1.81, which is the upper limit of the immersion liquids available for such experiments. The value of 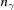 was calculated from the maximum birefringence, 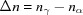, measured using Ehringhaus compensator on a Jenapol polarizing microscope. Crystals of (II) show extreme values of birefringence, 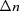 = 0.46 (2). The large value of 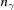 (1.91) can be attributed to a mutual arrangement of violurate and tyraminium ions in (II) causing a large resultant dipole moment responsible for the observed anisotropy of optical properties.

### 3.3. Electron-density distribution, atomic and bond polarizabilities   

The QTAIM analysis was focused on electron-density distribution of violuric acid and violurate ions as the change in their electronic state in the crystal structure was assumed to be responsible for the observed colour change in both the solid state and solution (Awadallah *et al.*, 1994 ▸). The topological descriptors at bond critical points (BCPs) found in (I), (II), (III) and VA for the oxime group atoms are presented in Table 2 ▸, whereas a full description of the bonds can be found in Tables S7 and S8. Geometrical analysis of bond lengths suggests that there are differences in the oxime group in all three structures. The longest N2A—O1A bond (1.349 Å) is present in the crystal structure of VA, where the oxime group of violuric acid is protonated. On the other hand, among the violurate ions this bond is significantly shorter in the range 1.260–1.282 Å. The deprotonation of an oxime group also affects the C5A—N2A bond, which, as expected, is longer in violurate ions. The small discrepancies in bond lengths are also observed for C5A—C6A and C5A—C4A bonds. The shortest C—C bonds are present in (II) and the longest in VA. There are no significant changes observed for other bonds forming the violurate ring. High values of ellipticity (>0.24) for C5A—N2A suggest double-bond character (Bader, 2003 ▸). The Laplacian profiles along the selected bond paths (C—N, N—O of the oxime group and C—C bonds of the violurate ring) remain similar when moving from a molecule to an ion (see Fig. S11). A slight shift of BCP positions in the N—O bond can be observed when moving from a molecule to an ion, which can be associated with the deprotonation of the O1A oxygen atom.

**Figure 8:**
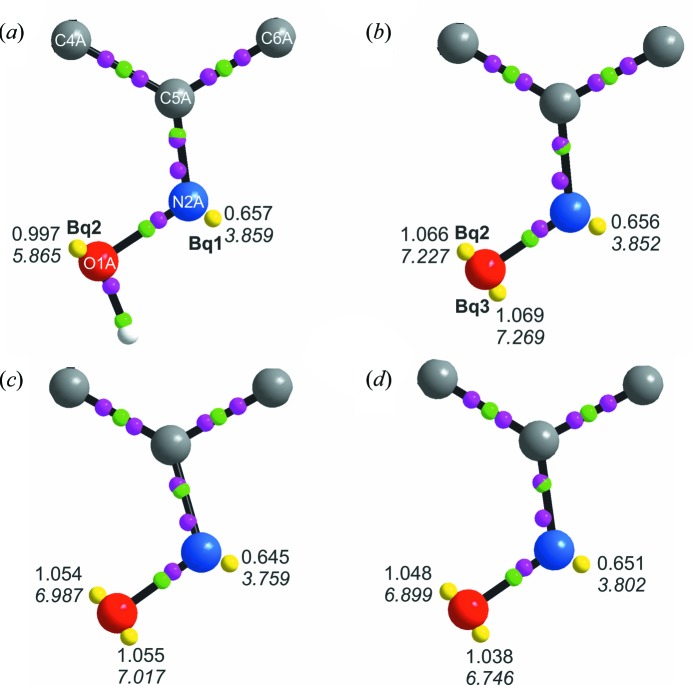
Laplacian CPs for (*a*) VA, (*b*) (II), (*c*) (III) and (*d*) (I). The values of charge density and negative Laplacian (shown in italic type) are given for all VSCC maxima. Small pink spheres represent VSCC maxima and small green spheres represent BCPs.

Surprisingly, there are no differences in the BCPs position in the oxime group within the three ions. Awadallah *et al.* (1994 ▸) suggested that mixing of the *n*-orbital of oxime nitro­gen atom with the lone pair of the oxime oxygen atom would influence the energy of the *n*–π* transition which is correlated directly with the colour change. To confirm that claim we have studied subtle changes in the electronic state of the atoms using valence-shell-charge concentrations (VSCCs) and source-function contribution to VSCC. This analysis enabled further examination of the impact of weak interactions on the density distribution within the oxime group. The negative Laplacian critical points of (3,−3) type associated with charge accumulation maxima were analysed for atoms within all three structures. As expected, the bonding maxima (BM) are placed along the bond paths and the nonbonding maxima (NBM) are situated near atomic positions (Fig. 8 ▸). Two BMs and one NBM can be found around the N2A atom supporting its *sp* ^2^ hybridization. The ρ(**r**) and Laplacian values of the N2A atom NBM in VA are comparable with the values in (I), (II) and (III). The presence of two NBMs for the oxime oxygen atom in (I) and (II) indicates an *sp* ^2^-hybridized O1A atom and a double N2A—O1A bond. A distinct description is obtained for the O1A atom in VA. Two NBMs for the oxime oxygen atom and its participation in two bonds prove an *sp* ^3^-hybridized O1A atom and a single N2A—O1A bond. The values of the Laplacian at the oxygen NBMs are slightly different in all three structures: the smallest values of the Laplacian are for VA, whereas the highest values are observed for (II). Additionally, the lack of NBMs around C5A suggests either formation of a double C5A—N2A bond or delocalization of electrons towards neighbouring atoms.

The source-function contributions to VSCC maxima (*Bq*1, *Bq*2 and *Bq*3), representing lone pairs of N2A and O1A (see Fig. S12 and Table 3 ▸), reveal subtle changes in the redistribution of charge around the atoms of the oxime group. There is a non-negligible contribution of O1A atom electron density to *Bq*1 representing the lone pair of nitro­gen N2A. This value increases from 3.31% to above 4% when going from violuric acid to violurates, with the highest percentage for the polymorph (II).

A smaller contribution can be observed from the carbon C5A atom to the *Bq*1 – around 1.71% for violuric acid up to 1.57% for (I). The oxygen O1A lone pair(s) also have a small contribution from the N2A atom – 1.58% in violuric acid, increasing to 2.42% in violurate ions. Comparison between the *Bq* maxima in the coloured violurate ions shows small changes in the percentage contributions from O, N and C atoms.

Discrepancies between the examined structures can be seen in the formation of weak interactions. We have compared the overall strength of the hydrogen bonds formed using oxygen O5A (*E*_O_) and nitro­gen N2A acceptors (*E*_N_) in all three polymorphs (Table 4 ▸). The values show a clear distinction in their hydrogen-bonding accepting properties. The highest hydrogen-bond energy can be found for nitro­gen oxime atoms in (II) (−5.46 kcal mol^−1^). This value is comparable with the −2.39 kcal mol^−1^ found for (I). There are no hydrogen bonds involving N atoms as acceptors in the VA structure and in polymorph (III). In the case of the O5A oxygen atom the highest interaction energy is found for (I), whereas the smallest is found for VA. The non-negligible difference in *E*_sum_, between (I) and (II) (6.5 kcal mol^−1^) and between (I) and (III) (9.85 kcal mol^−1^), can be associated with the moderate hydrogen bond formed between the violuric ion and water molecules in (I).

**Figure 9:**
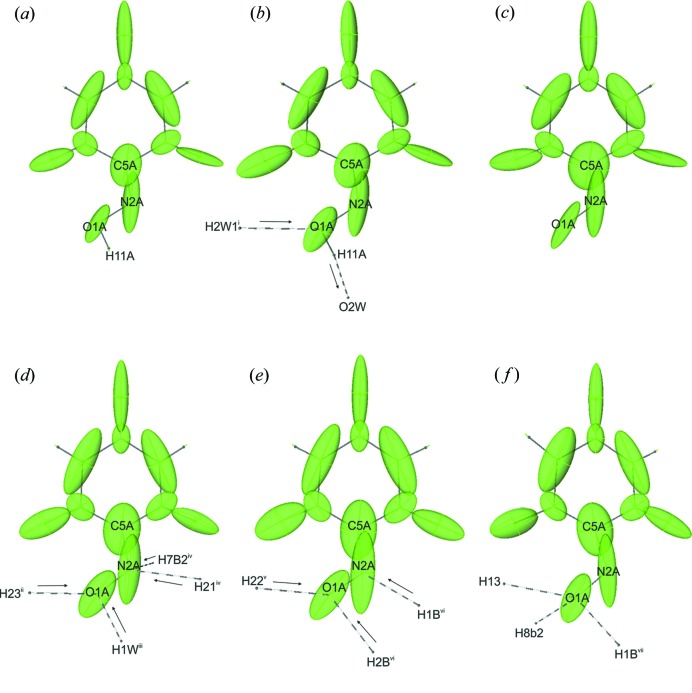
Distributed atomic polarizabilities of violuric acid (*a*) isolated, (*b*) taken from VA, (*c*) isolated ion, (*d*) taken from (I), (*e*) taken from (II), (*f*) taken from (III). The scaling factor is 0.4 Å^−2^. Dashed lines indicate interactions with the oxime group, arrows indicate the direction of the hydrogen bond from donor to acceptor. Symmetry codes: (i) −*x* + 1, −*y* + 1, *z* + 1/2; (ii) −*x* + 1/2, *y* − 1/2, −*z*; (iii) *x*, *y* − 1, *z*; (iv) −*x* + 1, −*y*, −*z*; (v) −*x*, *y* + 1/2, −*z* + 1/2; (vi) −*x*, −*y* + 1, −*z* + 1; and (vii) *x*, *y* + 1, *z*.

Those variations in the interaction energies do not correspond to the only minor fluctuations in electron densities at the N1A and O1A lone pairs. It is obvious that the formed hydrogen bonds are non-equivalent in strength and number, and the characteristics of the acceptor atoms (the amount of electron density available at the NBMs) should change because of this fact. In order to assess the impact of the weak interactions on the covalent bonds in the oxime group, atomic (Fig. 9 ▸) and bond (Table 5 ▸) polarizabilities were calculated. Bond polarizability, 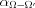, (Krawczuk *et al.*, 2014 ▸) reflects how much the electron density is polarized along a bond and is defined as 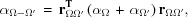
where 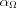 and 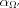 are atomic polarizabilities and 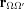 is a unit vector in the direction 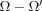 bond. Since the nitro­gen atom of the deprotonated oxime group in (II) is an acceptor of a stronger O—H⋯N hydrogen bond than in (I) (N—H⋯N), one could expect that the atomic polarizability of N2A should be more affected by the presence of the hydrogen bond and thus the C—N bond polarizability should be smaller. In fact we observe the opposite behaviour. The atomic polarizability of the nitro­gen atom in (I) is much more affected by the weaker bond than by the stronger bond in (II). The polarizability ellipsoid of N2A in (I) is flattened and slightly reoriented towards the donor group of the hydrogen bond, whereas in (II) it is prolated towards the C—N covalent bond [compare Figs. 9 ▸(*d*) and 9(*e*)].

Similar behaviour is observed when analysing the polarizability ellipsoids of the C5A atom. When moving from the isolated state [Fig. 9 ▸(*c*)] through violuric acid in (I) [Fig. 9 ▸(*d*)] towards the molecule in (II) [Fig. 9 ▸(*e*)], it is evident that the carbon atom polarizability ellipsoid changes from roll shape, through slightly prolated along the C—N bond, towards significantly elongated along that bond.

This behaviour together with the change in nitro­gen atomic polarizability causes the increase in the polarization of electron density along the C—N bond when moving from (I) to (II). After the phase transition from (I) to (III) there is a change in the bond polarizabilities, in particular the characteristics of the N—O bond become similar to those of VA, whereas the C—N bond remains similar to the one in (I). The obtained bond polarizabilities can be correlated with UV–Vis spectra in the solid state [see Fig. 6 ▸(*b*)], thus with the colour of a certain form. There is an evident interrelationship between the absorption-band wavelength, and the polarization of electron density in the oxime group, in particular along the C—N bond. The higher the absorption-band wavelength, the larger the bond polarizability along the C—N bond of an oxime group. One could also expect that for VA the absorption band should appear at shorter wavelength than for (I) [Fig. 6 ▸(*b*)], since the C—N bond polarizability is smaller than for (I) and (II) (compare also the behaviour of atomic polarizabilities in Fig. 9 ▸). It is interesting to correlate the interaction energies, net atomic charges and bond polarizabilities. Nitro­gen atom N2A does not participate as an acceptor of hydrogen bonds in VA and in (III), yet the C—N bond polarizabilities differ (Δα_C—N_ = 4.16). Net atomic charges for N2A, O1A and C5A in all structures show systematic changes (see Table S9). The decrease in positive charge of the carbon atom corresponds well with the decrease in negative charge on the adjacent N2A atom when going from VA to (I), (II) and (III). This clearly indicates that the change in colour is a co-operative process. In the case of violurates, the origin of colour is directly associated with the intrinsic rearrangement of the electron density in the C5A—N2A—O1A group – the deprotonation of the oxime group is responsible for the change of colourless violuric acid crystals to colourful violurates. However, the particular hue of the crystal can be tuned by changing the strength and directionality of intermolecular interactions as was performed in the case of tyraminium violurates.

## 4. Conclusions   

In this article we have examined the origin of colour in the organic violurates. Polymorphic and pseudopolymorphic forms of tyraminium violurate were chosen to study the interactions between the organic ions in the presence or absence of the solvent. Salts containing only one type of cation were selected to exclude the influence of the pH difference, between violuric acid and the base, on the colour formation. The combined theoretical and experimental studies (QTAIM, atomic and bond polarizabilities analyses) revealed that the origin of colour can be correlated with the change in the internal electron density distribution in the oxime group of violuric acid. The generation of colour is a two-step process including the proton transfer from violuric acid to a base (here tyramine) and the formation of the intermolecular interactions which are responsible for the final absorption spectrum of the new material. Our studies prove that the colour of the sample can be controlled by tuning intermolecular interactions in the solid state. This fact shows the large impact of directional interactions on the properties of materials and thus proves the usefulness of the crystal-engineering approach.

## Supplementary Material

Crystal structure: contains datablock(s) I, II, III. DOI: 10.1107/S2052252518017037/yc5016sup1.cif

Supporting tables and figures. DOI: 10.1107/S2052252518017037/yc5016sup2.pdf

Structure factors: contains datablock(s) I. DOI: 10.1107/S2052252518017037/yc5016sup3.hkl

Structure factors: contains datablock(s) II. DOI: 10.1107/S2052252518017037/yc5016sup4.hkl

Structure factors: contains datablock(s) III. DOI: 10.1107/S2052252518017037/yc5016sup5.hkl

Supporting information file. DOI: 10.1107/S2052252518017037/yc5016sup6.cml

Supporting information file. DOI: 10.1107/S2052252518017037/yc5016sup7.cml

CCDC references: 1860172, 1860173, 1860174

## Figures and Tables

**Table 1:** Experimental refractive indices (589 nm) and results of Q-LFT/MP2/6-311++G(d,p) calculations

		Refractive indices	
		Experiment	Theory
(I)	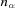	1.63	1.593
	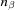	1.65	1.671
	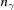	1.72	1.708
(II)	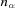	1.45	1.470
	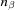	1.78	1.800
	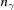	∼1.91†	1.911

†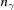 – calculated from maximum birefringence Δ*n* = 0.46 (2).

**Table 2:** Topological analysis of BCPs for selected bonds in VA (first row), (II) (second row), (III) (third row) and (I) (fourth row) *d*, intermolecular distance (Å); *d*1, *d*2, distance between BCPs and interacting atoms (Å); ρ(**r**), charge density (a.u.); ∇^2^ρ(**r**), Laplacian of electron density (a.u.); ∊, bond ellipticity.

Bond	*d*	*d*_1_	*d*_2_	ρ(**r**)	∇^2^ρ(**r**)	∊
N2A—O1A	1.349	0.624	0.725	0.328	−0.27	0.02
	1.260	0.588	0.672	0.406	−0.52	0.03
	1.279	0.595	0.684	0.392	−0.48	0.04
	1.282	0.599	0.683	0.386	−0.44	0.03
C5A—N2A	1.295	0.463	0.832	0.372	−1.06	0.31
	1.352	0.532	0.820	0.334	−1.02	0.24
	1.397	0.579	0.818	0.307	−0.80	0.24
	1.352	0.532	0.820	0.335	−1.01	0.27
C5A—C6A	1.478	0.725	0.753	0.280	−0.80	0.13
	1.438	0.686	0.752	0.299	−0.87	0.22
	1.447	0.688	0.759	0.294	−0.84	0.20
	1.456	0.698	0.758	0.290	−0.83	0.19
C5A—C4A	1.485	0.733	0.752	0.275	−0.78	0.12
	1.438	0.689	0.749	0.296	−0.86	0.20
	1.449	0.706	0.743	0.288	−0.83	0.19
	1.459	0.707	0.752	0.285	−0.81	0.18

**Table 3:** Source-function contributions to VSCC maxima showing the change in electron density

	*Bq*1 (%)			*Bq*2 (%)		*Bq*3 (%)	
	N2A	O1A	C5A	N2A	O1A	N2A	O1A
VA	92.87	3.31	1.71	1.58	96.61	—	—
(I)	92.98	4.24	1.57	2.29	97.00	2.19	96.51
(II)	91.87	4.56	1.48	2.42	96.50	2.32	96.53
(III)	92.27	4.14	1.48	2.17	96.75	2.26	96.66

**Table 4:** The sum of weak-interaction energies involving N2A and O1A atoms as acceptors of hydrogen bonds

	*E*_N_ (kcal mol^−1^)	*E*_O_ (kcal mol^−1^)	*E*_sum_
VA	0.00	−7.24	−7.24
(I)	−2.39	−20.41	−22.81
(II)	−5.46	−10.85	−16.31
(III)	0.00	−12.96	−12.96

**Table 5:** Bond polarizabilities for C5A–N2A and N2A–O1A in the studied compounds compared with values obtained for isolated molecules of violuric acid Values are given in Bohr^3^. iso1 is an isolated molecule of violuric acid with an N–O–H group. iso2 is an isolated molecule of violuric acid with a deprotonated oxime group.

	iso1	VA	iso2	(I)	(II)	(III)
C—N	29.71	29.33	34.01	33.68	37.18	33.49
N—O	20.49	22.35	25.86	26.18	26.88	22.55
