# Supplementary material for: Origin of chromic effects and crystal-to-crystal phase transition in the polymorphs of tyraminium violurate
Source: IUCrJ. 2019 Jan 24;6(Pt 2):226–37. doi: 10.1107/S2052252518017037 (PMC6400187; doi:10.1107/S2052252518017037)
Supplement: Supplementary file 2 [file m-06-00226-sup2.pdf]

IUCrJ

Volume 6 (2019)

Supporting information for article:

The origin of chromic effects and crystal-to-crystal phase transition in the polymorphs of tyraminium violurate

Marlena Gryl, Agnieszka Rydz, Joanna Wojnarska, Anna Krawczuk, Marcin Kozieł, Tomasz Seidler, Katarzyna Ostrowska, Monika Marzec and Katarzyna Marta Stadnicka

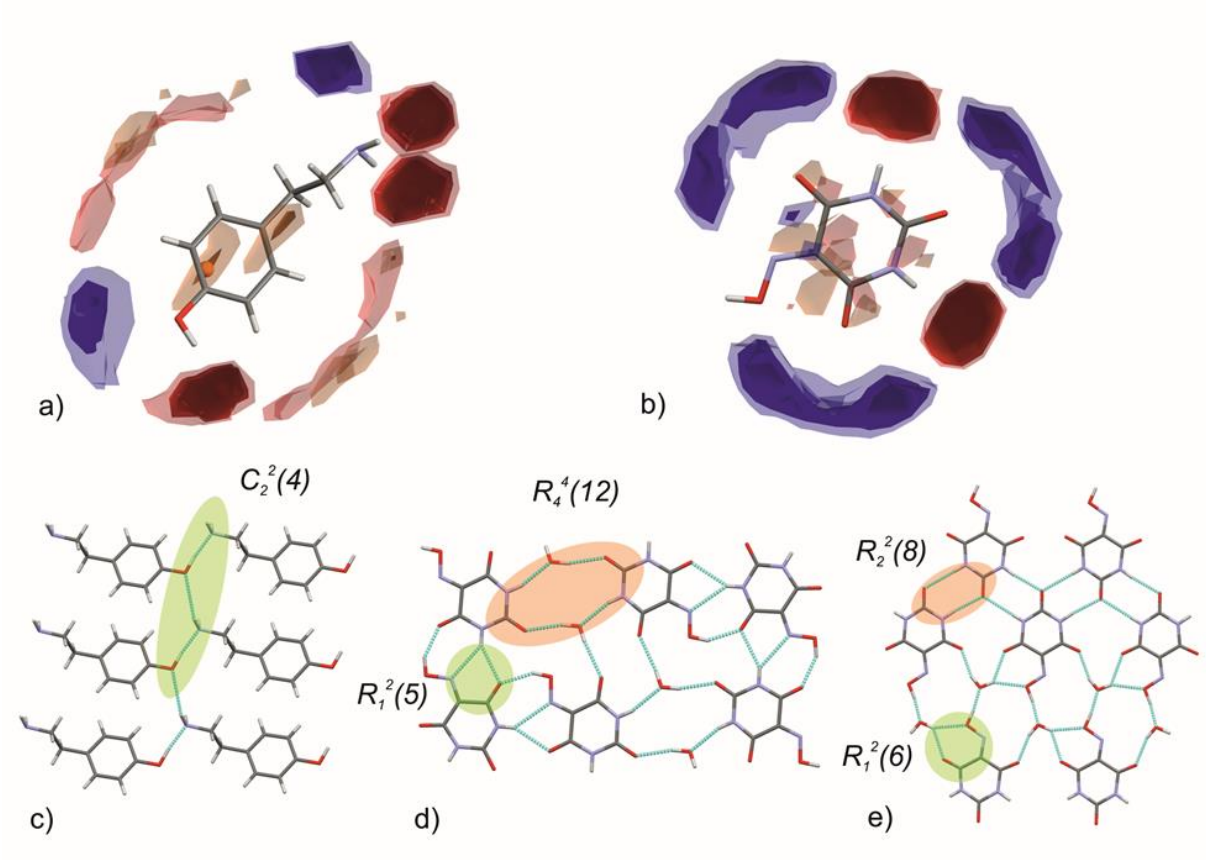

Figure S1 Full interaction maps of a) TYR and b) VA (P21/n). Red colour denotes possible donor sites, whereas blue - possible acceptor sites. Hydrogen-bond patterns in crystal structure of c) TYR ($P\bar{1}$), and d) VA monohydrate polymorph I (P21/n), and e) VA polymorph II (Cmc21).

Table S1 Data collection and refinement details of (I), (II) and (III) crystal structures.

	**(I)**	**(II)**	**(III)**
**Crystal data**			
Chemical formula	$C_{12}H_{16}N_4O_6$	$C_{12}H_{14}N_4O_5$	$C_{12}H_{14}N_4O_5$
M_r [g/mol]	312.29	294.27	294.27
Crystal system, space group	monoclinic, $P2_1/c$	monoclinic, $P2_1/c$	monoclinic, $P2_1/c$
Temperature [K]	130(2)	130(2)	130(2)
a, *b*, *c* (Å)	14.0525(3), 7.81340(10), 12.8242(2)	6.8711(1), 15.119(2), 11.884(2)	13.2706(1), 7.6510(3), 13.1269(9)
β (°)	91.848(2)	91.858(2)	102.097(8)
V [Å^3]	1407.34(4)	1233.9(4)	1303.22(16)
Z	4	4	4
Radiation type	Mo *K*α	Mo *K*α	Cu *K*α
V [Å^3]	1407.34(4)	1233.9(4)	1303.22(16)
Z	4	4	4
Density [g/cm^3]	1.474	1.584	1.500
μ (mm^{-1})	0.120	0.126	1.013
Crystal size [mm]	0.25 x 0.20 x 0.05	0.18 x 0.18 x 0.10	0.30 x 0.20 x 0.10
**Data collection**			
Diffractometer	Rigaku Oxford Diffraction SuperNova Dual Source diffractometer with an Atlas detector		
Absorption correction	multi-scan		
	CrysAlis PRO 1.171.38.34a (Rigaku Oxford Diffraction, 2015) Empirical absorption correction using spherical harmonics, implemented in SCALE3 ABSPACK scaling algorithm.		
T_{min}, T_{max}	0.894, 1.000	0.260, 1.000	0.608, 1.000
No. of measured, independent and observed [$I > 2\sigma(I)$] reflections	33526, 3819, 2992	23617, 4891, 2109	27510, 2661, 1678

R_{int}	0.0453	0.1779*	0.0863
(sin θ/λ)$_{max}$ (Å^{-1})	0.696	0.700	0.625
**Refinement**			
$R[F^2 > 2\sigma(F^2)]$, $wR(F^2)$, S	0.0400, 0.0888, 1.052	0.0673, 0.1472, 0.875	0.0809, 0.2118, 1.054
No. of reflections	3819	4891	2661
No. of parameters	4	0	6
No. of restraints	244	209	227
H-atom treatment			
$\Delta\rho_{max}$, $\Delta\rho_{min}$ (e Å^{-3})	0.349, -0.241	0.295, -0.310	0.393 and -0.265

*Crystals of (II) were twinned. The two components were separated using CrysAlisPro (version 1.171.36.20).

Table S2 Values of refractive indices and corresponding immersion liquids ratio for (I) and (II) crystals.

Crystal structure	Value of Refractive Index	Immersion Liquids Ratio
	1.63	50 μl of bromoform + 25 μl of methyl iodide
**(I)**	1.65	100 μl of bromoform + 75 μl of methyl iodide
	1.72	50 μl of bromoform + 275 μl of methyl iodide
	1.45	150 μl of xylene
**(II)**	1.77	supersaturated solution of sulfur in methyl iodide
	1.91	birefringence measurements

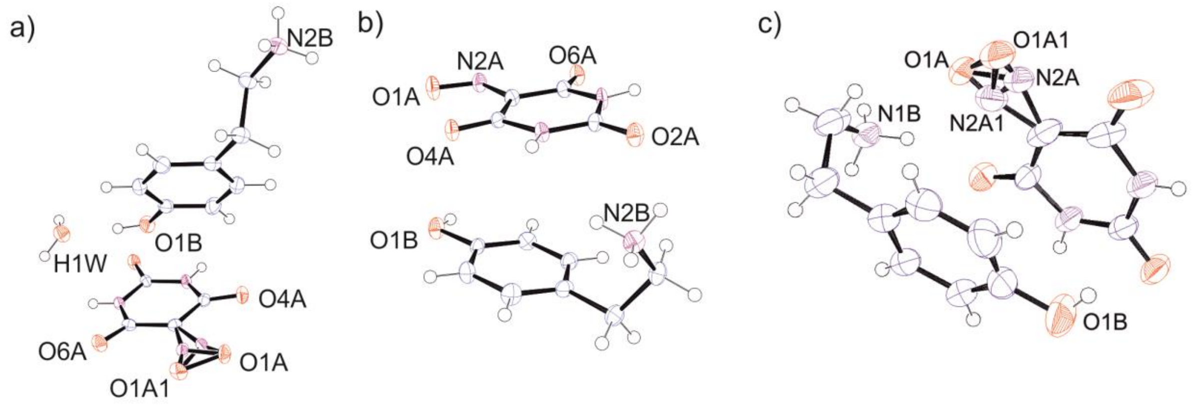

Figure S2 Contents of the asymmetric units of a) (I), b) (II), and c) (III) crystal structure.

Table S3 Hydrogen-bonds geometry in crystal structure of (I).

D-H···A	Distance D-H [Å]	Distance H···A [Å]	Distance D-A [Å]	∢ D-H···A [Å]
O1W-H1W···O1A1$^{(i)}$	0.86(9)	1.76(1)	2.590(5)	161(1)
O1B-H1B···O1W$^{(ii)}$	0.82(9)	1.83(9)	2.625(1)	164(1)
O1W-H1W···O1A$^{(i)}$	0.86(9)	1.87(1)	2.661(1)	152(1)
N1A-H1A···O2A$^{(iii)}$	0.89(1)	1.91(1)	2.805(1)	177(1)
N3A-H3A···O6A$^{(ii)}$	0.84(1)	2.06(1)	2.831(1)	153(1)
N2B-H23···O1A$^{(iv)}$	0.90(1)	2.07(1)	2.759(1)	133(1)
N2B-H23···O4A$^{(iv)}$	0.90(1)	2.18(1)	2.896(1)	136(1)
N2B-H21···O1A1$^{(v)}$	0.92(1)	2.08(1)	2.768(4)	131(9)
O1W-H2W···O4A$^{(vi)}$	0.84(1)	2.09(1)	2.870(1)	155(2)
N2B-H21···O6A$^{(v)}$	0.92(1)	2.12(1)	2.889(1)	141(1)
N2B-H22···O1B$^{(vii)}$	0.92(1)	2.157(1)	3.037(1)	159(1)

(i) x, y-1, z; (ii) x, -y-1/2, z-1/2; (iii)-x+1, -y-1, -z+1, (iv) -x, y-1/2, -z+1/2, (v) -x, -y, -z+1; (vi) x, -y-1/2, z+1/2; (vii) -x, y+1/2, -z+1/2.

Table S4 Hydrogen-bonds geometry in crystal structure of (II).

D-H···A	Distance D-H [Å]	Distance H···A [Å]	Distance D-A [Å]	∢ D-H···A [Å]
N1A-H1A···O4A^{i}	0.93(3)	1.93(4)	2.834(4)	163(3)
N3A-H3A···O6A^{ii}	0.86(4)	1.99(4)	2.848(4)	172(3)
O1B-H1B···N2A^{iii}	0.86(4)	2.05(4)	2.870(4)	161(3)
N2B-H22···O4A^{iv}	0.84(4)	2.07(4)	2.758(4)	139(3)
N2B-H23···O6A^{ii}	0.96(4)	2.16(4)	2.991(4)	143(3)
N2B-H22···O1A^{iv}	0.84(4)	2.19(4)	2.847(4)	135(3)
N2B-H21···O2A^{v}	0.94(4)	2.34(4)	3.046(4)	132(3)
C2B-H2B···O1A^{iii}	0.95	2.45	3.339(4)	155

(i) x,-y+1/2,z+1/2; (ii) x,-y+1/2,z-1/2; (iii) -x,-y+1,-z+1; (iv) -x,y-1/2,-z+1/2; (v) -x,-y,-z+1

Table S5 Hydrogen-bonds geometry in crystal structure of (III).

D-H···A	Distance D-H [Å]	Distance H···A [Å]	Distance D-A [Å]	∢ D-H···A [Å]
O(1B)-H(1B)...O(2A2)$^{(i)}$	0.80(2)	1.73(4)	2.336(11)	131(5)
N1A-H1A···O2A$^{(ii)}$	0.90(2)	1.93(2)	2.824(4)	171(4)
N(2B)-H(22)...O(4A)$^{(iii)}$	0.86(1)	1.95(2)	2.750(4)	155(4)
O(1B)-H(1B)...O(2A1)$^{(i)}$	0.80(2)	2.11(3)	2.863(8)	157(5)
N(2B)-H(23)...O(6A)	0.86(1)	2.13(3)	2.905(5)	149(4)
N(2B)-H(21)...O(1B)$^{(iv)}$	0.86(1)	2.14(3)	2.919(5)	150(4)
N3A-H3A···O6A$^{(v)}$	0.89(2)	2.20(2)	3.069(5)	165(4)
N(2B)-H(23)...O(2A1)	0.86(1)	2.26(4)	2.841(7)	124(4)
C6B-H6B···O4A$^{(iv)}$	0.95	2.40	3.042(8)	121
C8B-H8B2···O2A1	0.99	2.42	3.040(8)	121

(i)x, y-1, z; (ii) -x+2, -y, -z+2 (iii) x, -y+3/2, z-1/2; (iv) x, -y+1/2, z-1/2 (v) x, -y+1/2, z-1/2.

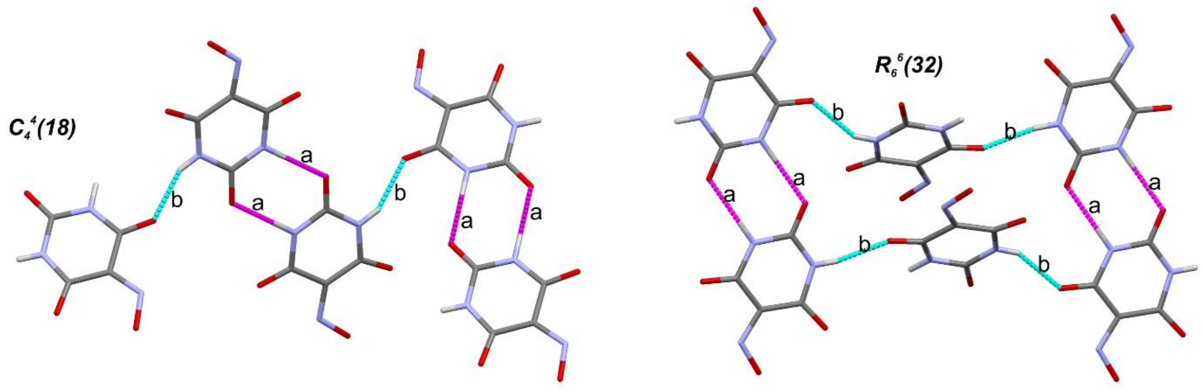

Figure S3 Representative hydrogen-bond motifs of (I).

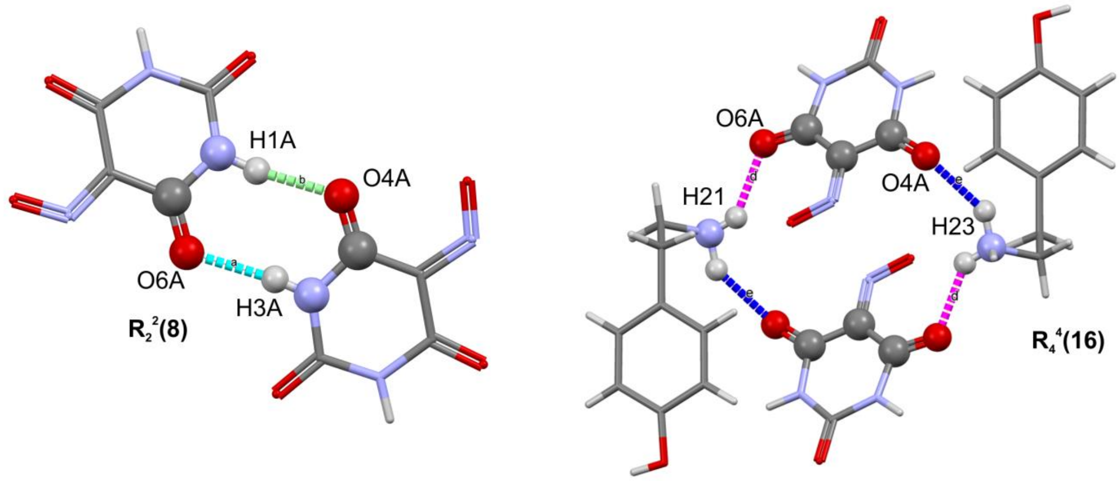

Figure S4 Representative hydrogen-bond motifs of (II).

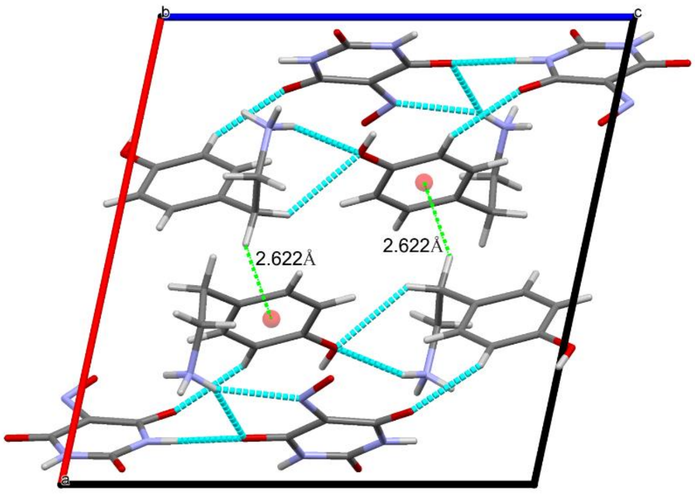

Figure S5 C-H···π interactions in (III) between tyraminium layers.

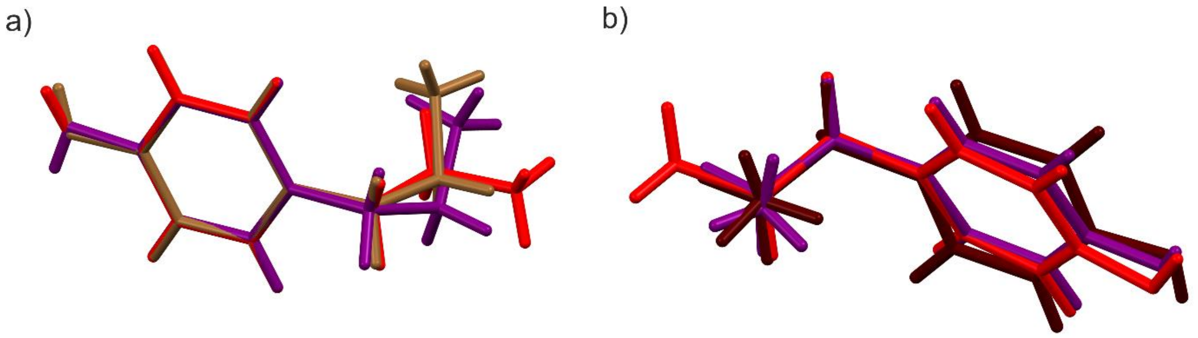

Figure S6 Comparison of conformation of tyraminium cations in crystal structure of (I) – red, (II) – violet and (III) –brown: a) molecules overlay using aromatic rings b) molecules overlay using C4B-C7B-C8B moiety.

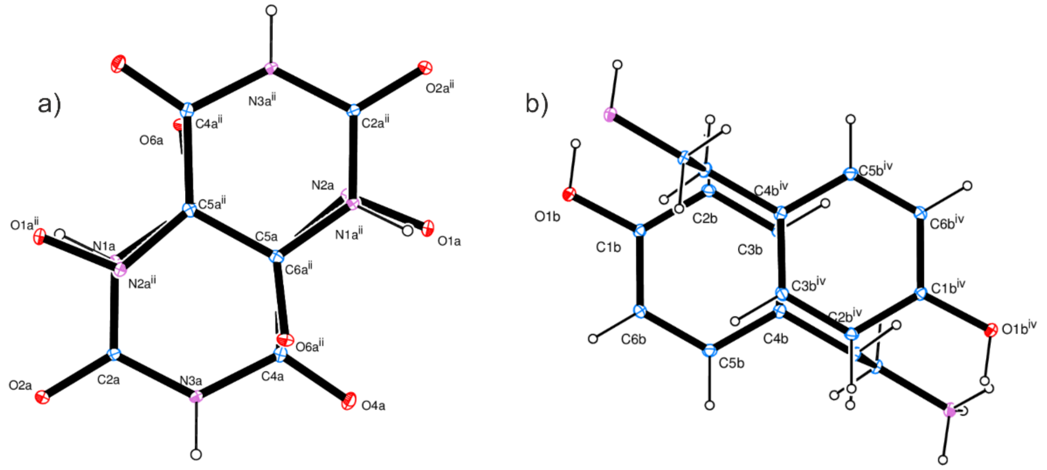

Figure S7 Form I a) Ct1-Ct1i = 3.915 Å. Projection onto the violurate ring plane (N1A^C6A), b) Ct2-Ct2i = 4.361 Å. Projection onto the tyraminium ring plane (C1B^C6B). In both cases there are no pi-pi interactions.

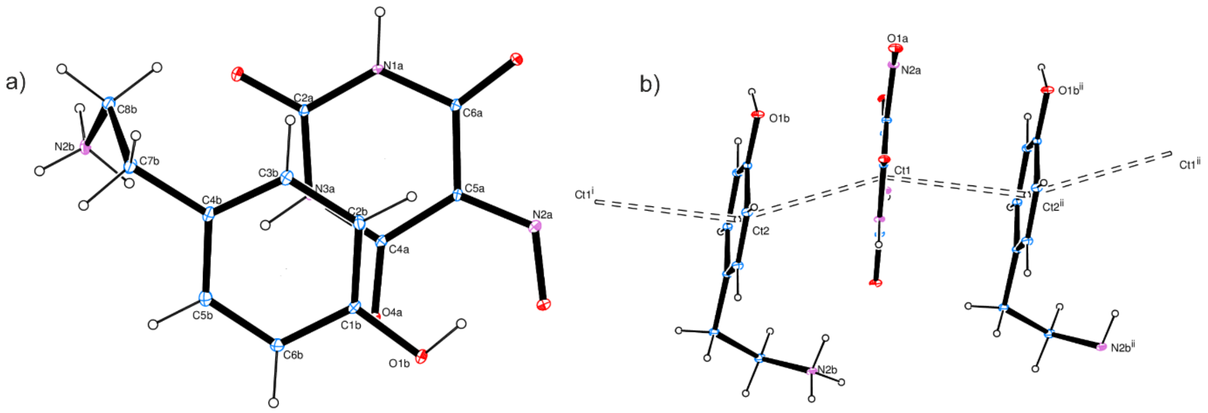

Figure S8 Form II: a) Ct1-Ct2 = 4.055 Å . Projection onto the violurate ring plane (N1A^C6A) b) Ct1-Ct2 = 4.055 A and Ct1-Ct2(x-1, y, z) = 4.104 Å. Side view of the mutual position of the violurate (N1A^C6A) and the tyraminium (C1B^C6B) rings. Note that the rings are not parallel. In both cases there are no pi-pi interactions.

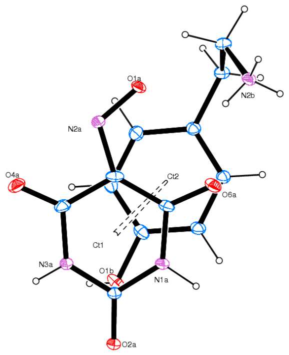

Figure S9 Form III: Ct1-Ct2 = 3.812 Å . Projection onto the violurate ring plane (N1A^C6A) shows that there is no pi-pi interaction between the rings and the rings are not parallel.

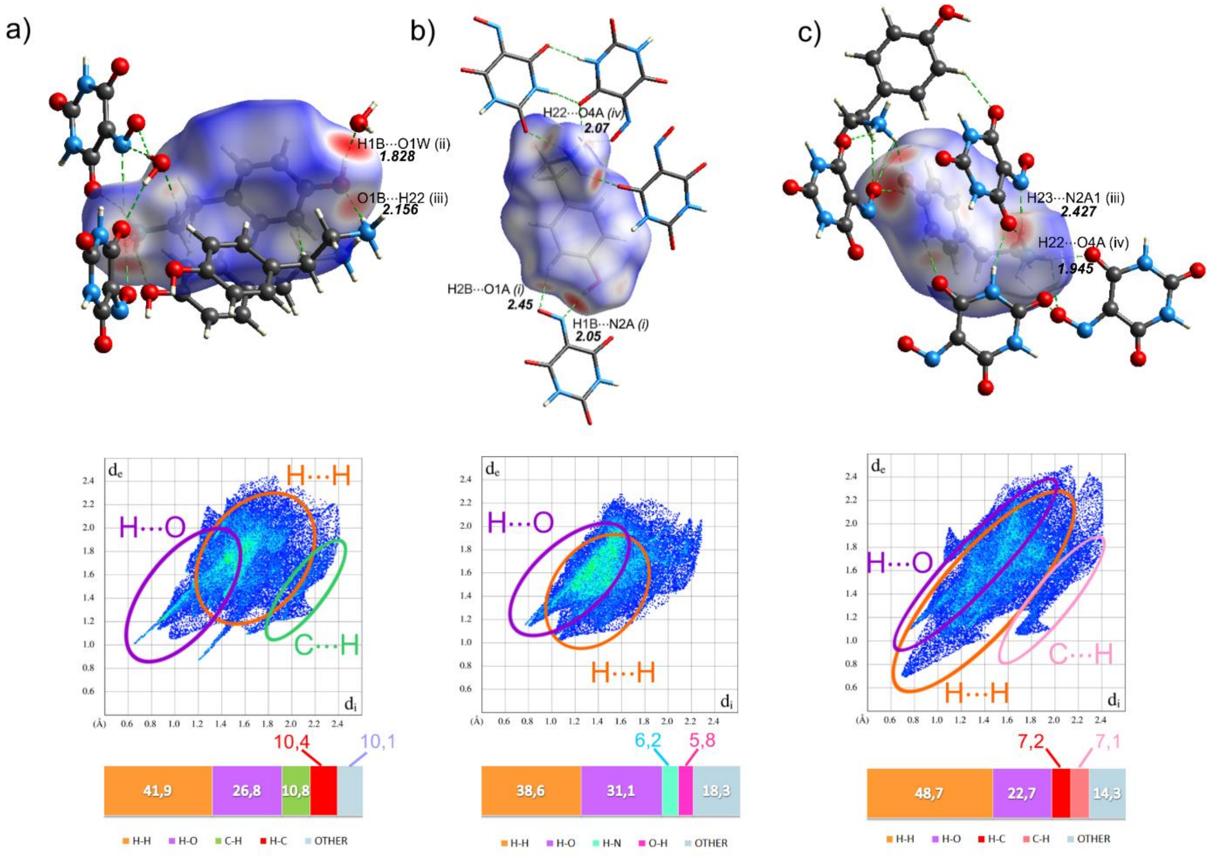

Figure S10 Hirshfeld surfaces (top) and 2-D fingerprint plots (bottom) for tyraminium cation in crystal structure of a) (I) b) (II) and c) (III).

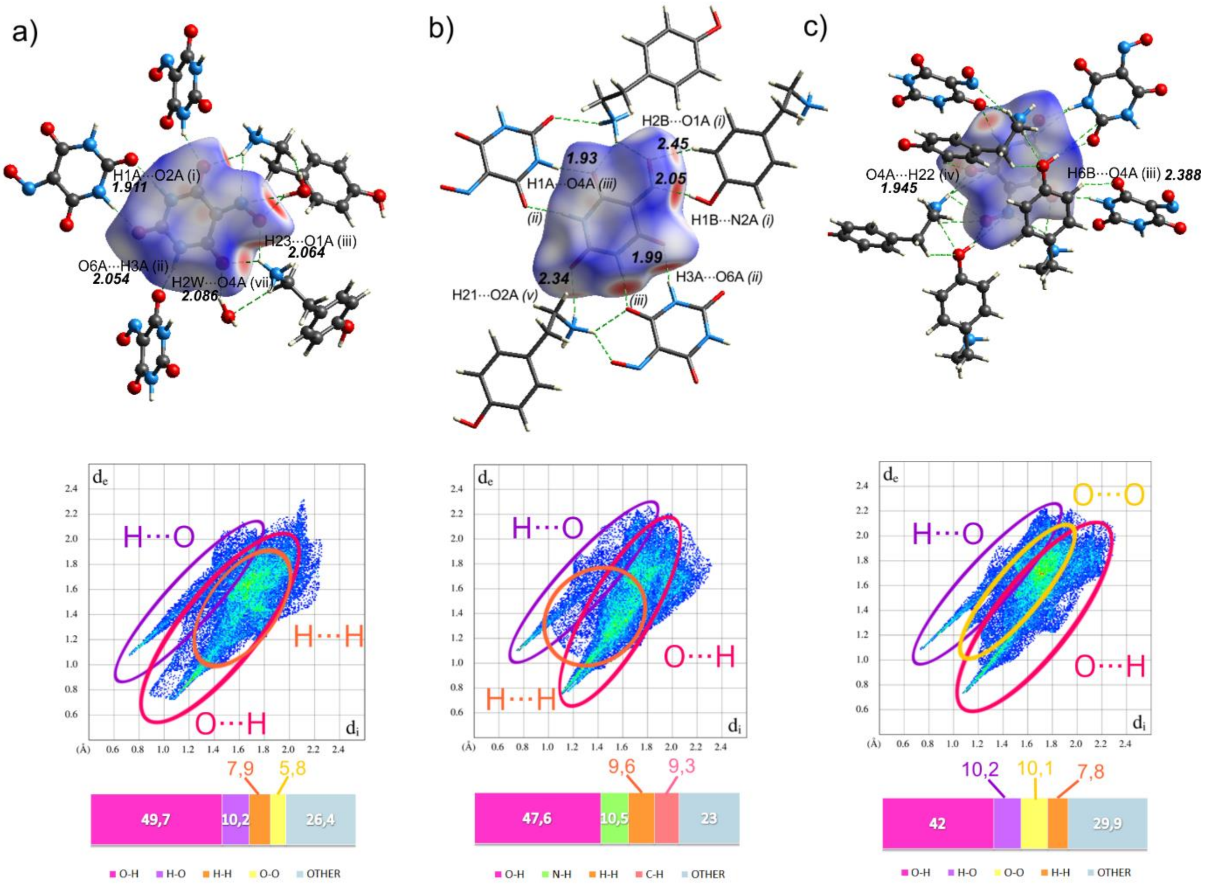

Figure S11 Hirshfeld surfaces (top) and 2-D fingerprint plots (bottom) generated for violurate anion in a) (I) b) (II), and c) (III).

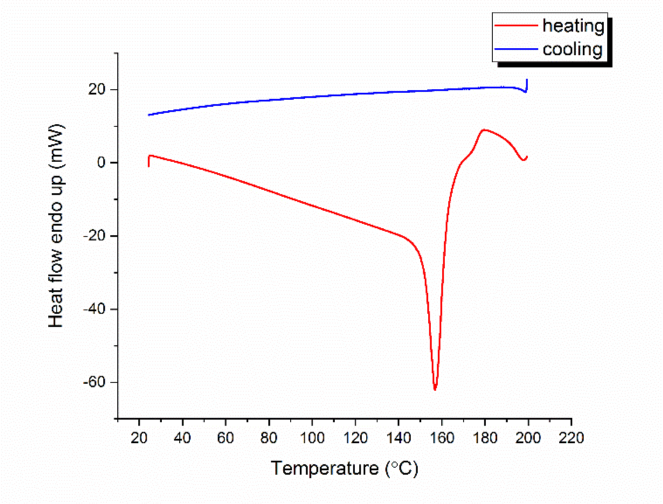

Figure S12 DSC curves for the crystals of (I).

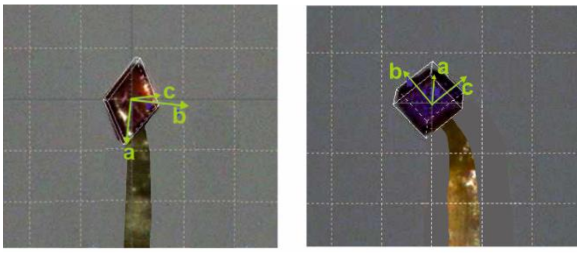

Figure S13 Orientation of crystallographic axes for (I) and (II), respectively.

Table S6 Theoretical calculations of refractive indices of (I) and (II) crystals (a and b orientations correspond to one of the two conformations of disordered oxime group).

	**λ/nm**	**n_α**	**n_β**	**n_γ**
**(I) – orientation a**	∞	1.550	1.611	1.652
	1064	1.559	1.621	1.667
	659	1.577	1.637	1.695
	532*	1.599	1.652	1.724
**(I) – orientation b**	∞	1.541	1.628	1.639
	1064	1.549	1.641	1.650
	659	1.566	1.666	1.670
	532*	1.587	1.690	1.691
**(I) - averaged**	∞	1.546	1.625	1.640
	1064	1.554	1.636	1.654
	659	1.572	1.654	1.681
	532*	1.593	1.671	1.708
**(II)**	∞	1.451	1.761	1.847
	1064	1.456	1.775	1.869
	659	1.473	1.800	1.911
	532*	1.627	1.824	1.959

Table S7 Topological analysis of critical points for selected bonds in VA (first row), (II) (second row) (III) (third row) and (I) (4th row).

Bond	d	d_1	d_2	ρ(r)	$\nabla^2\rho(r)$	λ_1	λ_2	λ_3	ε
N2A- O1A	1.349	0.624	0.725	0.328	-0.27	-0.72	-0.71	1.17	0.02
	1.260	0.588	0.672	0.406	-0.52	-0.96	-0.93	1.37	0.03
	1.279	0.595	0.684	0.392	-0.48	-0.92	-0.89	1.34	0.04
	1.282	0.599	0.683	0.386	-0.44	-0.90	-0.87	1.32	0.03
C5A-N2A	1.295	0.463	0.832	0.372	-1.06	-0.88	-0.68	0.50	0.31
	1.352	0.532	0.820	0.334	-1.02	-0.76	-0.61	0.35	0.24
	1.397	0.579	0.818	0.307	-0.80	-0.68	-0.55	0.42	0.24
	1.352	0.532	0.820	0.335	-1.01	-0.76	-0.60	0.35	0.27
C5A-C6A	1.478	0.725	0.753	0.280	-0.80	-0.61	-0.54	0.35	0.13
	1.438	0.686	0.752	0.299	-0.87	-0.66	-0.54	0.33	0.22
	1.447	0.688	0.759	0.294	-0.84	-0.65	-0.54	0.34	0.20
	1.456	0.698	0.758	0.290	-0.83	-0.64	-0.53	0.34	0.19
C5A-C4A	1.485	0.733	0.752	0.275	-0.78	-0.59	-0.53	0.34	0.12
	1.438	0.689	0.749	0.296	-0.86	-0.65	-0.54	0.33	0.20
	1.449	0.706	0.743	0.288	-0.83	-0.63	-0.53	0.33	0.19
	1.459	0.707	0.752	0.285	-0.81	-0.62	-0.53	0.34	0.18
C4A-O4A	1.209	0.414	0.795	0.423	-0.52	-1.12	-1.01	1.61	0.10
	1.227	0.422	0.805	0.406	-0.62	-1.04	-0.96	1.39	0.09
	1.219	0.418	0.801	0.414	-0.56	-1.09	-0.99	1.52	0.1
	1.227	0.422	0.805	0.407	-0.63	-1.05	-0.96	1.38	0.09
C6A-O6A	1.209	0.414	0.795	0.423	-0.51	-1.12	-1.02	1.63	0.09
	1.233	0.425	0.808	0.401	-0.69	-1.01	-0.94	1.27	0.07
	1.228	0.423	0.805	0.406	-0.64	-1.04	-0.96	1.35	0.08
	1.233	0.425	0.808	0.402	-0.68	-1.02	-0.95	1.29	0.07
C6A-N1A	1.386	0.545	0.841	0.306	-0.93	-0.69	-0.62	0.37	0.11
	1.388	0.545	0.843	0.305	-0.92	-0.68	-0.62	0.37	0.10
	1.398	0.547	0.851	0.296	-0.87	-0.65	-0.59	0.37	0.09
	1.375	0.533	0.842	0.313	-0.98	-0.70	-0.64	0.36	0.10
C4A-N3A	1.383	0.543	0.84	0.308	-0.94	-0.69	-0.62	0.37	0.12
	1.387	0.544	0.843	0.306	-0.93	-0.68	-0.62	0.37	0.10
	1.395	0.556	0.839	0.302	-0.89	-0.67	-0.6	0.38	0.12
	1.387	0.548	0.839	0.306	-0.93	-0.69	-0.61	0.37	0.12
C2A-N3A	1.367	0.544	0.823	0.323	-1.06	-0.76	-0.66	0.37	0.15
	1.355	0.533	0.822	0.331	-1.10	-0.79	-0.67	0.36	0.17
	1.367	0.541	0.826	0.323	-1.05	-0.76	-0.66	0.37	0.16
	1.370	0.549	0.821	0.322	-1.03	-0.76	-0.65	0.38	0.17

C2A-N1A	1.378	0.558	0.820	0.318	-1.00	-0.75	-0.64	0.39	0.16
	1.359	0.537	0.822	0.329	-1.08	-0.78	-0.67	0.36	0.17
	1.361	0.551	0.81	0.33	-1.07	-0.78	-0.67	0.38	0.18
	1.370	0.551	0.819	0.323	-1.03	-0.76	-0.65	0.38	0.17
C2A-O2A	1.224	0.423	0.801	0.415	-0.76	-1.09	-0.97	1.30	0.13
	1.223	0.423	0.800	0.415	-0.76	-1.10	-0.98	1.32	0.12
	1.232	0.427	0.805	0.408	-0.81	-1.06	-0.95	1.2	0.12
	1.228	0.425	0.803	0.412	-0.78	-1.08	-0.96	1.25	0.12

Table S8 Topological analysis of critical points for selected bonds in VA (first row), (II) (second row) (III) (third row) and (I) (4th row).

Bond	ρ(r)	$\nabla^2\rho(r)$	G(r)	V(r)	E(r)	|V(r)|/G(r)	G(r)/ρ(r)	E(r)/ρ(r)
N2A- O1A	0.328	-0.27	0.208	-0.483	-0.275	2.32	0.634	-0.838
	0.406	-0.52	0.283	-0.695	-0.413	2.46	0.697	-1.017
	0.392	-0.48	0.267	-0.652	-0.385	2.44	0.681	-0.982
	0.386	-0.44	0.263	-0.637	-0.374	2.42	0.681	-0.969
C5A-N2A	0.372	-1.06	0.357	-0.979	-0.623	2.74	0.960	-1.675
	0.334	-1.02	0.196	-0.645	-0.449	3.29	0.587	-1.344
	0.307	-0.80	0.150	-0.501	-0.351	3.34	0.489	-1.143
	0.335	-1.01	0.200	-0.652	-0.452	3.26	0.597	-1.349
C5A-C6A	0.280	-0.80	0.063	-0.327	-0.265	5.20	0.225	-0.946
	0.299	-0.87	0.081	-0.380	-0.30	4.70	0.271	-1.003
	0.294	-0.84	0.082	-0.373	-0.292	4.56	0.279	-0.993
	0.290	-0.83	0.075	-0.357	-0.282	4.77	0.259	-0.972
C5A-C4A	0.275	-0.78	0.061	-0.315	-0.255	5.18	0.222	-0.927
	0.296	-0.86	0.08	-0.375	-0.295	4.69	0.270	-0.997
	0.288	-0.83	0.074	-0.354	-0.281	4.79	0.257	-0.976
	0.285	-0.81	0.071	-0.345	-0.275	4.87	0.249	-0.965
C4A-O4A	0.423	-0.52	0.643	-1.415	-0.772	2.20	1.520	-1.825
	0.406	-0.62	0.574	-1.301	-0.727	2.27	1.414	-1.791
	0.414	-0.56	0.609	-1.356	-0.748	2.23	1.471	-1.807
	0.407	-0.63	0.574	-1.304	-0.731	2.27	1.410	-1.796
C6A-O6A	0.423	-0.51	0.645	-1.416	-0.771	2.20	1.525	-1.823
	0.401	-0.69	0.545	-1.261	-0.716	2.31	1.359	-1.786
	0.406	-0.64	0.568	-1.296	-0.728	2.28	1.399	-1.793
	0.402	-0.68	0.55	-1.269	-0.72	2.31	1.368	-1.791
C6A-N1A	0.306	-0.93	0.157	-0.546	-0.39	3.48	0.513	-1.275
	0.305	-0.92	0.156	-0.542	-0.387	3.48	0.511	-1.269

	0.296	-0.87	0.153	-0.523	-0.371	3.42	0.517	-1.253
	0.313	-0.98	0.17	-0.585	-0.415	3.44	0.543	-1.326
C4A-N3A	0.308	-0.94	0.159	-0.552	-0.394	3.48	0.516	-1.279
	0.306	-0.93	0.158	-0.548	-0.391	3.47	0.516	-1.278
	0.302	-0.89	0.146	-0.513	-0.368	3.52	0.483	-1.219
	0.306	-0.93	0.152	-0.536	-0.385	3.53	0.497	-1.258
C2A-N3A	0.323	-1.06	0.156	-0.576	-0.42	3.69	0.483	-1.300
	0.331	-1.10	0.173	-0.62	-0.447	3.58	0.523	-1.350
	0.323	-1.05	0.161	-0.585	-0.424	3.64	0.498	-1.313
	0.322	-1.03	0.154	-0.565	-0.411	3.67	0.478	-1.276
C2A-N1A	0.318	-1.00	0.145	-0.539	-0.394	3.72	0.456	-1.239
	0.329	-1.08	0.168	-0.606	-0.438	3.61	0.511	-1.331
	0.330	-1.07	0.157	-0.580	-0.424	3.70	0.476	-1.285
	0.323	-1.03	0.152	-0.562	-0.41	3.70	0.471	-1.269
C2A-O2A	0.415	-0.76	0.567	-1.323	-0.756	2.33	1.366	-1.822
	0.415	-0.76	0.566	-1.322	-0.756	2.34	1.364	-1.822
	0.408	-0.81	0.534	-1.270	-0.736	2.38	1.309	-1.804
	0.412	-0.78	0.551	-1.296	-0.746	2.35	1.337	-1.811

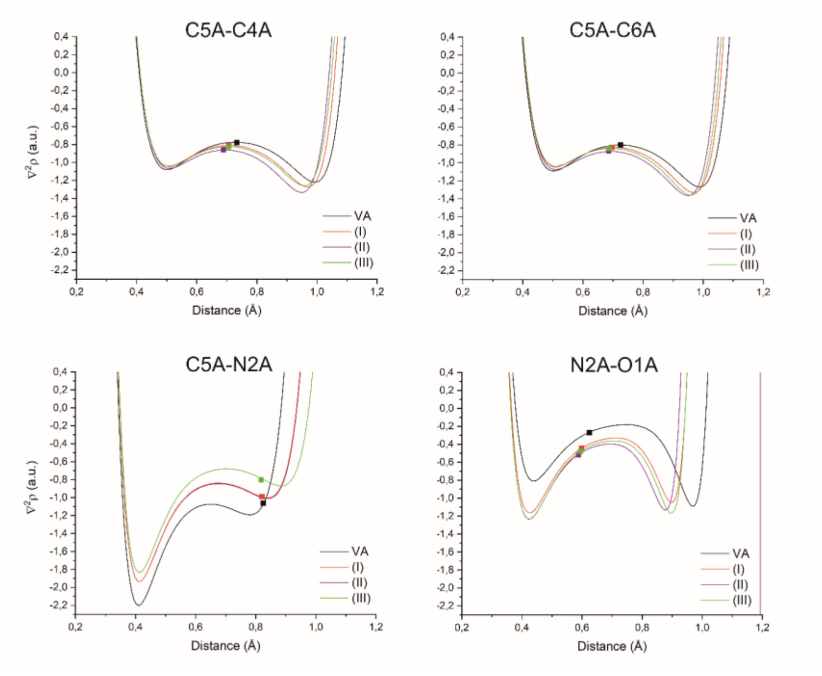

Figure S14 Laplacian profiles for C5A-C4A, C5A-C6A, C5A-N2A and N2A-O1A in violurate ions.

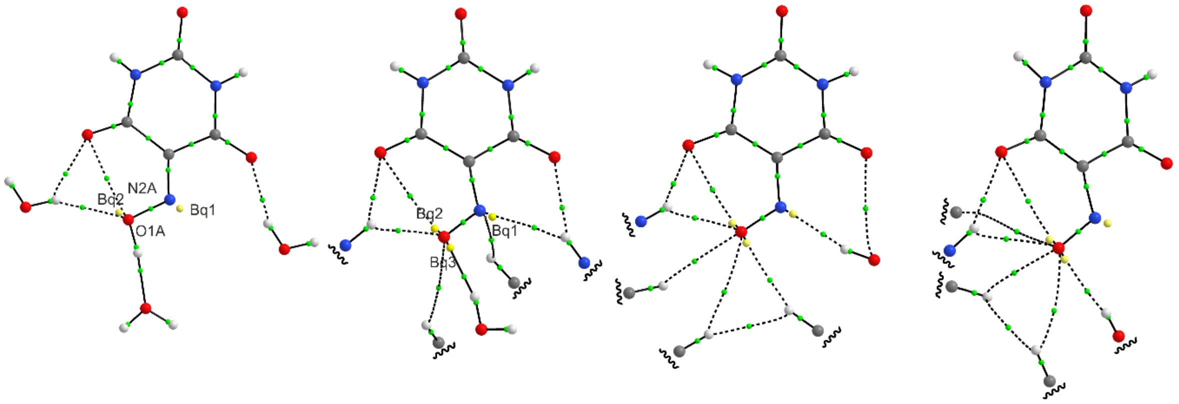

Figure S15 Intermolecular interactions in a) VA b) (I) c) (II) d) (III) with marked positions of bcp (green spheres). Yellow spheres (Bq) mark the position of VSCC maxima. Some atoms were omitted for clarity.

Table S9 Net atomic charges for nitrogen N2A, oxygen O1A and carbon atom C5A in VA, (I), (II) and (III).

	q_{C5A}	q_{N2A}	q_{O1A}
**VA**	+0.53	-0.39	-0.70
**(I)**	+0.37	-0.27	-0.63
**(II)**	+0.36	-0.24	-0.63
**(III)**	+0.31	-0.20	-0.66
